# The road to evolution of ProTx2: how to be a subtype-specific inhibition of human Na_v_1.7

**DOI:** 10.3389/fphar.2024.1374183

**Published:** 2024-05-02

**Authors:** Fan Zhao, Yuanyuan Liu, Yiyu Liu, Qi Ye, Hongtao Yang, Mingze Gui, Yongbo Song

**Affiliations:** School of Life Science and Biopharmaceutics, Shenyang Pharmaceutical University, Shenyang, China

**Keywords:** ProTx2, voltage-gated sodium ion channel, analgesia, structure optimization, toxin

## Abstract

The human voltage-gated sodium channel Na_v_1.7 is a widely proven target for analgesic drug studies. ProTx2, a 30-residue polypeptide from Peruvian green tarantula venom, shows high specificity to activity against human Na_v_1.7, suggesting its potential to become a non-addictive analgesic. However, its high sensitivity to human Na_v_1.4 raises concerns about muscle side effects. Here, we engineered three mutants (R13A, R13D, and K27Y) of ProTx2 to evaluate their pharmacological activities toward Na_v_1.7 and Na_v_1.4. It is demonstrated that the mutant R13D maintained the analgesic effect in mice while dramatically reducing its muscle toxicity compared with ProTx2. The main reason is the formation of a strong electrostatic interaction between R13D and the negatively charged amino acid residues in DII/S3-S4 of Na_v_1.7, which is absent in Na_v_1.4. This study advances our understanding and insights on peptide toxins, paving the way for safer, effective non-addictive analgesic development.

## 1 Introduction

Nociception, the body’s response to harmful stimuli, serves as a protective mechanism. However, when pain becomes chronic, it significantly diminishes the quality of life, escalates healthcare costs, and burdens society. In 2022, a social survey study in the United States highlighted that over 50 million adults suffered from chronic pain (Yong et al., 2022). In the ongoing battles against pain, voltage-gated sodium (Na_v_) channel 1.7, predominantly located in the peripheral nervous system, has emerged as a promising target for non-addictive development, owing to its integral role in pain signal generation and transmission (King and Vetter, 2014; Alsaloum et al., 2020; Jiang et al., 2021). The link between human Na_v_1.7 (hNa_v_1.7) dysfunction and diseases such as congenital insensitivity to pain (CIP), chronic pain syndromes, and inherited erythromelalgia (IEM) has been identified in loss-of-function and gain-of-function genetic studies (Cox et al., 2006; Fertleman et al., 2006). Therefore, the inhibitors targeting hNa_v_1.7 are considered to be the potential therapeutic target for non-addictive analgesics. However, the development of selective Na_v_1.7 inhibitors is challenging due to the high degree of similarity among sodium channel subtypes, (de Lera Ruiz and Kraus, 2015), which complicates specificity and heightens the risk of adverse effects.

**FIGURE 1:**
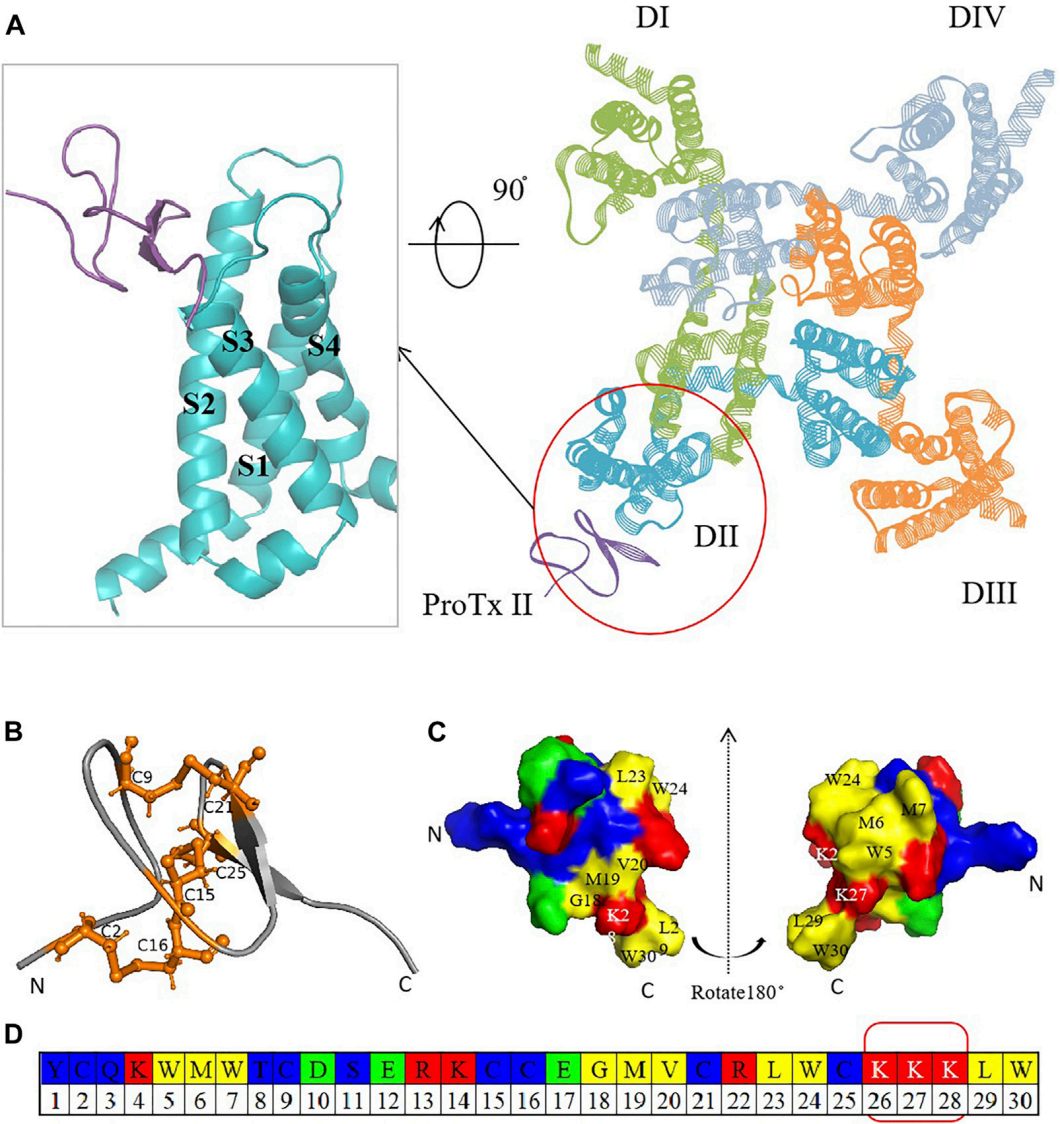
Crystal structure diagram of hNa_v_1.7 and ProTx2. **(A)** Complex of ProTx2 binding with hNa_v_1.7. The illustration on the left provides a close look at the ProTx2-binding area (PDB:6N4I). ProTx2 is highlighted in purple, and the four homologous domains of hNa_v_1.7 are shown in green, blue, yellow, and gray (in order to clarify the observation structure, some loop areas are removed). **(B)** Pairs of disulfide bonds in ProTx2; the residues that form the bonds are represented in orange. **(C)** 3D structure rotated at 180° to show the polybasic C-terminus and hydrophobic amino acids. **(D)** ProTx2 sequence (in Figures B and C, amino acids are represented in different colors: acidic: green, basic: red, hydrophilic: blue, and hydrophobic: yellow).

The mammalian Na_v_ channel, comprising one α-subunit and one or two auxiliary β-subunits, is the active site for drugs. The α-subunit consists of four homologous domains (DI-DIV) (Pless et al., 2014), with each domain consisting of six transmembrane α helices (S1–S6). These domains were split into two functional modules (Kaczmarski and Corry, 2014). S1–S4 form the voltage-sensing domain (VSD), and the conserved negatively charged residues in S4 enable its role, whereas four S5–S6 helices together form one pore domain (PD) surrounded by four VSDs (Payandeh et al., 2011; Noreng et al., 2021). Recent research has highlighted the voltage-sensing domain (VSD) as a more promising target for subtype-specific inhibitors due to its variability among subtypes (Huang et al., 2022). In 2018, Jian Payandeh et al. elucidated the structural basis of Na_v_1.7 regulation by the gating-modifier spider toxin Protoxin-II (ProTx2) via X-ray scattering technology and cryo-electron microscopy (Payandeh and Hackos, 2018). Their groundbreaking work significantly contributed to the development of selective peptide-based antagonists targeting Na_v_ (Figure 1A) (Xu et al., 2019).

Peptide-based drugs, characterized by their potent physiological activity, favorable oral bioavailability, and low immunogenicity, hold significant promise in targeting specific channels (Li et al., 2023). For Na_v_, numerous animal toxins have long been identified to bind to VSDs as natural modifiers (Leipold et al., 2005; Deuis et al., 2017; Xu et al., 2020). ProTx2, a 30-residue polypeptide derived from Peruvian green tarantula, stands out for its strong affinity to hNa_v_1.7 (IC_50_ at 0.3 nM), mediated by its unique structural features, including three disulfide bonds (C2–C16, C9–C21, and C15–C25) (Moore et al., 2012; Flinspach et al., 2017; Wu et al., 2018; Neff et al., 2020) and a polybasic C-terminal (K26–K27–K28) (Wright et al., 2017), which enhance its interaction with Na_v_ by an electrostatic attraction (Figures 1B–D). ProTx2 shows rapid voltage-dependent reversal for NaV1.5 channels, the primary sodium channel in the heart (Sokolov et al., 2008). A chimera model of Na_v_1.7 with ProTx2 published in 2019 provided more inspiration (Xu et al., 2019). Despite this promising potential, the challenge remains to mitigate ProTx2’s affinity for Na_v_1.4 (IC_50_ at 39 nM), which is crucial for skeletal muscle function, to prevent unwanted side effects such as myotonia or paralysis (Schmalhofer et al., 2008).

To address this, three single-point mutations (R13A, R13D, and K27Y) in ProTx2 were engineered to refine its specificity for hNa_v_1.7 and hNa_v_1.4, using a combination of computational and experimental approaches. The results indicated that the mutant R13D exhibited enhanced analgesic efficacy and selectivity for Na_v_1.7, positioning it as an encouraging candidate for pain treatment.

## 2 Materials and methods

### 2.1 Cell culture

Na_v_1.4-CHO cells and Na_v_1.7-CHO cells were maintained in Iscove’s modified Dulbecco’s medium (Gibco) supplemented with 10% (v/v) fetal bovine serum (FBS) and 200 μg/mL G418 (Sigma). All cells were cultured at 37 °C and 5% CO_2_ atmosphere. Mouse myoblasts C2C12, obtained from the Stem Cell Bank of The Chinese Academy of Sciences, were grown in Dulbecco’s modified Eagle‘s medium (DMEM) enriched with 10% heat-inactivated FBS, 2 mM L glutamine, 100 U/mL of penicillin, and 100 μg/mL of streptomycin. The C2C12 cells typically reached confluence in 3–5 days in 75-T flasks. For passaging, monolayers were treated with 0.25% trypsin for 3–5 min, rinsed twice with DMEM, and sub-cultured at a ratio of 1:4. Similarly, the Chinese hamster ovary (CHO) cells were cultured in IMDM medium supplemented with 10% fetal bovine serum, 1% penicillin/streptomycin, 100 μM hypoxanthine, and 16 μM thymidine. When 90% confluence was achieved, cells were separated by 0.25% trypsin EDTA, and patch clamp electrophysiology experiments were carried out.

### 2.2 Protein expression and purification

**FIGURE 2:**
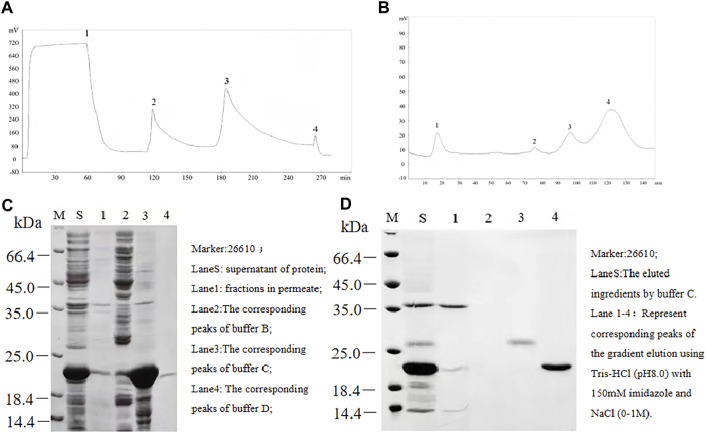
Separation and purification of ProTx2 fusion protein. **(A)** ProTx2 fusion protein profiles of nickel chelation affinity chromatography. **(B)** 12% SDS-PAGE analysis of each eluted ingredient in A. **(C)** ProTx2 fusion protein profiles of Q Sepharose Fast Flow chromatography. **(D)** 12% SDS-PAGE analysis of each eluted fraction in C.

The ProTx2 gene and its mutants (101 bp) were cloned into the pET-32a vector, incorporating TrxA, 6xHis, and enterokinase cleavage sites. Agarose gel electrophoresis confirmed the presence of the expected 100-bp bands, indicating successful plasmid construction (Supplementary Figure S1). Sequencing confirmed 100% amino acid fidelity with the original sequence, affirming the successful creation of the active peptides (Supplementary Figure S2). IPTG (isopropyl β-D-1-thiogalactopyranoside) induction yielded prominent expression bands for both ProTx2 and its mutants, aligning with the expected molecular weight of approximately 21 kDa (Figure 2). Subsequent analysis revealed over 90% expression of the target protein in the supernatant post-cell lysis, confirming effective soluble expression in the pET-32a system. Purification via nickel chelate affinity chromatography (buffer A: Tris-HCl, pH 8.0; buffer B: Tris-HCl with 50 mM imidazole, pH 8.0; buffer C: Tris-HCl with 150 mM imidazole, pH 8.0; and buffer D: 0.05M EDTA) and subsequent SDS-PAGE analysis identified a clear band corresponding to the ProTx2 fusion protein at 21 kDa, indicative of successful elution (Figures 2C,D). Further purification was achieved by Q Sepharose Fast Flow ion-exchange chromatography, employing a gradient elution strategy (gradient elution, the high-concentration eluent was Tris-HCl with 150 mM imidazole and 1M NaCl, pH 8.0; the low-concentration eluent was Tris-HCl with 150 mM imidazole, no salt) to isolate the target protein effectively. Enterokinase was finally utilized to cleave the fusion of His tags. Protein purity was assessed using Tricine–SDS-PAGE and quantified by HPLC analysis with area normalization (Supplementary Figures S3A, S3B).

### 2.3 Sensory assays

Male KM mice (18–22 g) were obtained from Liaoning Changsheng Biotechnology Co., Ltd. The mice were acclimated for 1 week at 18°C–22°C and 50%–70% humidity, following approval by the Animal Ethics Committee of Shenyang Pharmaceutical University, China. The pain response was assessed using a formalin-induced paw-licking test. The saline solution, 5 mg/kg morphine, commonly used analgesic medications in clinical practice, or 0.75 mg/kg peptides, ProTx II, and its mutants, were injected via the tail vein to relief the pain. After half an hour, a 20-μL 5% (v/v) formalin solution was administered as an activator to induce pain in mice (n = 10) (Miranda et al., 2007). The cumulative pain behavior that occurred 0–5 min (phase I, an immediate nociceptive phase) and 20–60 min (phase II, a later inflammatory phase) after injection was recorded for 60 min at 5 min intervals in individual cages to measure the total licking time in phases I and II, respectively.

Additionally, a forced swimming test measured endurance following 7 days of treatment with ProTx2 or its mutants (Porsolt et al., 1977; Galeotti et al., 2002; Xu et al., 2017). Fifty male KM mice were randomly divided into five groups with 10 mice in each group. The groups were classified as normal saline control, ProTx2, R13A, R13D, and K27Y.

### 2.4 Toxicity assays

C2C12 cell viability in response to ProTx2 or it mutants was evaluated using the CCK8 assay (Pan et al., 2019; Ge et al., 2020). A set of concentration gradients ranged from 0, 20, 40, 60, 80, 100, 120, 140, and 160 μg/mL, using a serum-free medium as a diluent. Cells were seeded in 96-well plates with 5*10^3^/well in five duplicates. After 18 h of treatment, cell viability was assessed by measuring the absorbance at 450 nm, following the addition of the CCK-8 reagent.

### 2.5 Molecular dynamics simulations

The simulation utilized human-derived PDB structures 6AGF (hNa_v_1.4) and 6N4I (hNa_v_1.7), with lipid bilayers modeled using POPC in Charmm-GUI (https://charmm-gui.org/) (Chen and Chung, 2012). Simulations were performed using Amber 18, employing the Amber-ff14SB force field (Maier et al., 2015), with the particle mesh Ewald (PME) algorithm for electrostatics (Pederson and McDaniel, 2022) and the SHAKE algorithm (Kräutler et al., 2001) for hydrogen movement. Langevin software maintained the temperature, with a 2-fs time step over a 100-ns simulation. Energy decomposition was conducted with MM/GBSA to analyze binding energies and key residue contributions (Izaguirre et al., 2001; Walton and Vanvliet, 2006).

### 2.6 Energy decomposition analysis

After 100-ns MD, the root mean square deviation (RMSD) and cluster analysis were performed to determine the quality of simulation by the CPPTRAJ (Roe and Cheatham III, 2013). RMSD curves showed the volatility of atoms in the athletic systems whose stationary band represented a stable simulation trajectory. Cluster analysis was used to identify the similarity of poses in the simulation from the stable trajectory. hieragglo epsilon was set as 1.5 Å, and the backbone atoms were calculated. For the analysis, the structures would be steady if the largest cluster occupied a proportion greater than 50% in all clusters.

Energy decomposition analysis (EDA) was carried out to analyze the binding energy between ProTx2 and Na_v_1.7/Na_v_1.4 in order to detect the ability of the combination of different ligands and receptors and show the contribution of every residue in ligands. The molecular mechanics generalized Born surface area (MM/GBSA) method (Hou et al., 2011) was performed on the basis of the snapshots extracted from the stable period of the trajectories. The total binding energy would be calculated, which was the overall evaluation for a combination including the sum of average molecular mechanical gas phase energies (EMM) and salvation-free energies (Gsolv). In more detail, EMM was divided into the summation of van der Waals (Evdw) and electrostatics (Eele) interaction energies, and Gsolv was described as a polar and non-polar solvation interaction due to an implicit solvent model and being solvent-accessible. Meanwhile, the decomposition of binding free energy of each ligand–residue pair emerged from which the key residues with high energy contribution (lower than −1 kcal/mol) were selected, and their dominant interactions were detected.

### 2.7 Dynamic cross-correlation analysis

Dynamic cross-correlation analysis (Hünenberger et al., 1995) (DCCM) was performed to the last stable trajectories of MD to speculate the relative region in the dynamic combination between ligands and receptors using CPPTRAJ of Amber20. The coordinate matrix was calculated based on the coordinates of Cα on the complexes which were set as the input data. The relevancy in the dynamic simulation will be analyzed between the residues from the construction of the former matrix results.

In addition, the salt-bridge, one kind of a strong electrostatic force, was calculated using VMD 1.9.2 (Humphrey et al., 1996), which was regarded as existent when the distance was less than 4 Å with a proportion higher than 50%. The hydrogen bond was analyzed using CPPTRAJ in Amber20, whose criterion was also 50% in all snapshots. Other parameters in the methods were kept as default.

### 2.8 Electrophysiology

Automatic electrophysiological platforms (NPC-1 and Port-a-Patch systems, Nanning Technologies GmbH, Munich, Germany) and EPC10 amplifiers (HEKA Elektronik, Lambrecht, Germany) assessed the effects of recombinant ProTx2 and its mutants on CHO-Na_v_1.4 and CHO-Na_v_1.7. PatchControl HT (Nanion Technologies GmbH, Germany) and PatchMaster software applications (HEKA Elektronik, Lambrecht, Germany) were used to execute the experimental process.

Cells underwent separation using 0.25% trypsin, followed by centrifugation at 800 rpm for 5 min, and were subsequently resuspended in extracellular fluid. CHO cells displaying characteristic morphology and vitality under microscopic examination were selected, and 10 cells were grouped per set. Microelectrodes were fashioned from blank glass tubes through a two-step pulling process, resulting in tip diameters of 0.8–1 μm and resistance levels between 1 and 3 MΩ. The composition of the extracellular solution was as follows (in mol/L): 140 NaCl, 4 KCl, 5 D-glucose monohydrate, 1 MgCl_2_, 2 CaCl_2_, and 10 HEPES (N-2-hydroxyethylpiperazine-N-2-ethane sulfonic acid)/NaOH, adjusted to a pH of 7.4 and maintained at 4°C. The intracellular electrode solution contained the following (in mol/L): 50 CsCl, 10 NaCl, 60 CsF, 20 EGTA (Xu et al., 2021), and 10 HEPES/CsOH, with a pH of 7.2. Both solutions were filtered through a 0.22-μm filter and stored at −20°C (Xiangxue et al., 2015; Xu et al., 2020).

In our whole-cell recordings, potassium currents were inhibited using Cs and a relatively high Mg^2+^ concentration (2 mmol/L). Cells exceeding 35 μm in diameter were exclusively examined, with a holding potential set at −80 mV. Sodium currents were elicited with a test pulse from −80 to +80 mV, in increments of 10 mV over 50-ms intervals (Xu et al., 2017). The transient sodium current amplitudes in CHO cells both before and subsequent to the addition of ProTx2 and R13D were documented. All experimental procedures were conducted at an ambient temperature of 22°C.

### 2.9 Data analysis

Statistical analyses were conducted using SPSS 22.0, employing one-way ANOVA and Tukey’s *post hoc* tests for group comparisons (Shenoy et al., 2021). Results were considered significant at *p* < 0.05. GraphPad Prism 5, Pymol (Schiffrin et al., 2020) and Gunplot (Latendresse et al., 2022) generated kinetic plots from molecular dynamic simulation data.

## 3 Results

### 3.1 Recombinant expression purification and identification of ProTx2

ProTx2 gene and its mutants (101 bp) were successfully cloned into the pET-32a vector, incorporating TrxA, 6xHis, and enterokinase cleavage sites (Figure 2).

Agarose gel electrophoresis confirmed the presence of the expected 100-bp bands, indicating successful plasmid construction (Figure 2) (Supplementary Figure S2).

IPTG induction yielded distinct expression bands of approximately 21 kDa, matching the anticipated sizes of the fusion proteins (Figure 2).

Subsequent purification via nickel chelate affinity chromatography and Q Sepharose Fast Flow ion-exchange chromatography resulted in a prominent band corresponding to the ProTx2 fusion protein at 21 kDa. Enterokinase cleavage and ultrafiltration provided a purified ProTx2 sample, with a final purity of approximately 96%, as determined by HPLC (Figures 2C,D) (Supplementary Figure S3B).

### 3.2 The enhanced analgesic effect of R13D mutants

**FIGURE 3:**
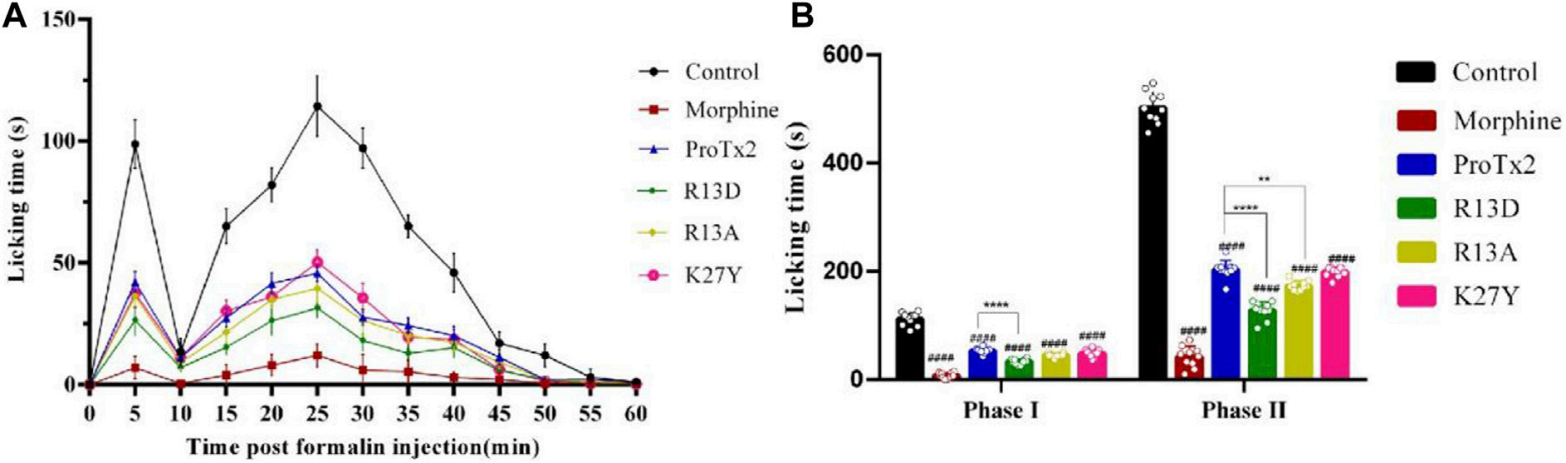
Analgesic effect of ProTx2 and mutants in the formalin model. **(A)** Time course of the analgesic effects of ProTx2 and mutants. **(B)** Evaluation of the antinociceptive effects of ProTx2 and mutants on phases I and II. (####*p* < 0.0001 *versus* control, **p < 0.01, ****p < 0.0001 *versus* wild ProTx2).

Three potential hNa_v_1.7 subtype-specific ProTx2 mutants (R13D, R13A, and K27Y) were identified through a virtual screen. The formalin-induced paw-licking assay in mice was established, and the cumulative pain behavior was recorded for 60 min at 5-min intervals (Figure 3A). The total licking time in phase I (0–5 min) and phase II (20–60 min) was measured, respectively, (Figure 3B). In phase I, the durations of total licking time of the control, morphine, wild ProTx2, R13D, R13A, and K27Y groups were 112.3 ± 3.9 s, 7.5 ± 1.6 s, 53.3 ± 1.6 s, 33.5 ± 1.6 s, 45.5 ± 1.1 s, and 48.7 ± 2.4 s, respectively, while in phase II, they were 502.6 ± 9.3 s, 42.1 ± 6.1 s, 203.5 ± 5.3 s, 128.0 ± 5.2 s, 173.5 ± 2.8 s, and 199.1 ± 2.9 s, respectively. These results revealed that the R13D mutant significantly alleviated pain in both the acute and inflammatory phases, outperforming the wild-type ProTx2. Specifically, R13D exhibited a marked reduction in licking time during both pain phases, suggesting an improved analgesic profile.

### 3.3 The reduced muscular toxicity of ProTx2 mutants

**FIGURE 4:**
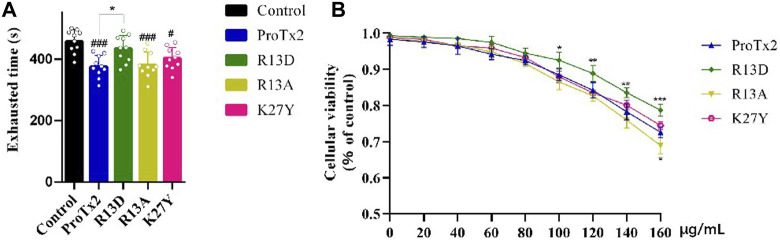
Validation of muscle toxicity. **(A)** Swimming time of ProTx2 and mutants in a forced swimming test (n = 10). **(B)** Cellular viability of C2C12 with ProTx2 and mutants (n = 5). (*p* < 0.001, #*p* < 0.05 *versus* control, *p < 0.05, **p < 0.01, ***p < 0.001 *versus* wild ProTx2).

Following a 7-day treatment regimen, mice received injections of ProTx2 or its mutants and underwent forced swimming tests 20 min after the final treatment. Notably, R13D-treated mice exhibited significantly prolonged endurance compared to the wild-type ProTx2 group, suggesting reduced muscular toxicity (*p* < 0.05). In contrast, the performance of mice treated with R13A and K27Y did not significantly differ from those treated with wild-type ProTx2 (Figure 4).

Additionally, to elucidate the interaction between the peptide’s toxicity and Nav1.4, the viability of C2C12 myoblasts, which express Nav1.4 abundantly, after 18-h exposure to varying concentrations of ProTx2 and its mutants were assessed. Remarkably, R13D demonstrated significantly improved cellular viability in a concentration-dependent manner, particularly between 100 and 160 μg/mL, underscoring its reduced toxicity relative to the wild-type peptide. No significant differences in cellular viability were observed with R13A and K27Y, aligning with the forced swimming test outcomes.

### 3.4 Molecular dynamics reveal key residue contributions to ProTx2 specificity

**FIGURE 5:**
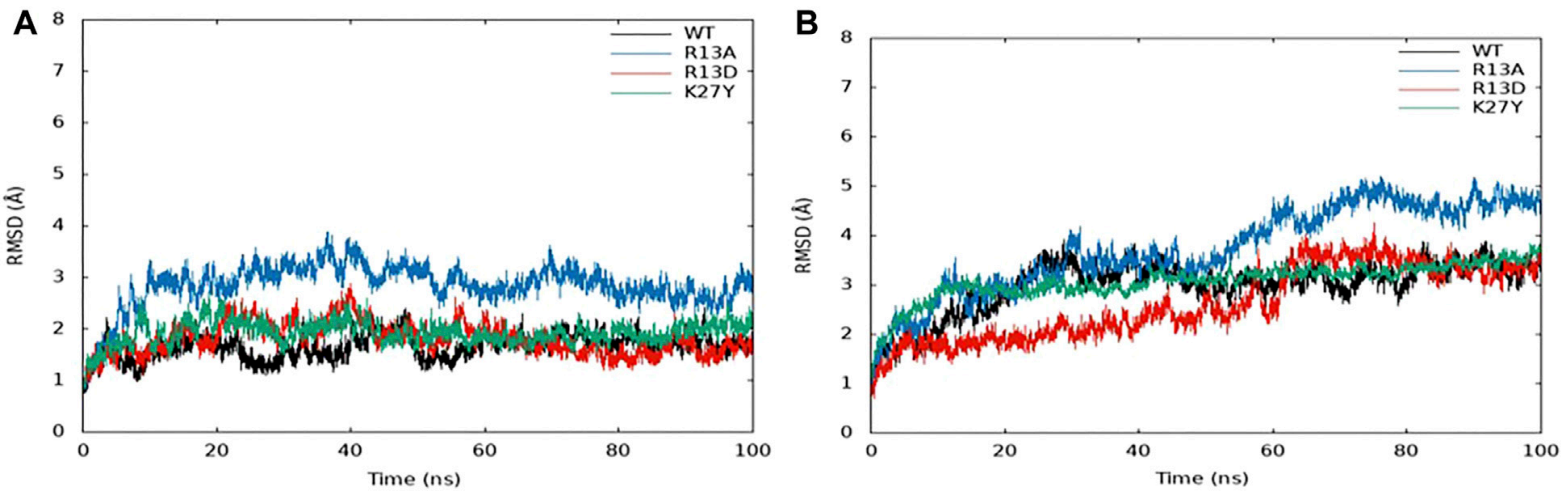
RMSD of the ProTx2 complex system: **(A)** hNa_v_1.7. **(B)** hNa_v_1.4.

Molecular dynamic (MD) simulations over 100 ns provided detailed insights into the interactions between ProTx2 and its mutants (R13D, R13A, and K27Y) with hNav1.7 and hNav1.4 (Figure 5). Root mean square deviation (RMSD) analyses indicated that with the exception of K27Y, both wild-type ProTx2 and the other mutants bound similarly to hNav1.7, suggesting minimal alterations in their binding modes. However, notable conformational changes were observed when these peptides interacted with hNav1.4, particularly for R13D.

Dynamic cross-correlation matrix (DCCM) analyses of stable simulation trajectories (90–100 ns) further highlighted the specific interactions driving these effects (Piao et al., 2019). Enhanced interactions between the peptides’ C-terminal (residues 160–170) and hNav1.7’s extracellular loop (DII S3–S4, residues 99–108) were evident post-mutation, as marked by intensified correlation signals (red box in Supplementary Figure S4). Similarly, changes were noted at the peptides’ N-terminal and the extracellular loop connecting S1–S2 at DII (residues 39–48) on hNav1.7 (brown box in Supplementary Figure S4).

In contrast, the interaction dynamics between R13D and hNav1.4’s extracellular loops showed significantly strengthened negative correlations, suggesting a distinct modulation of binding post-mutation, which is not observed with R13A or K27Y. These findings align with the structural data from a published chimera model (Xu et al., 2019), confirming that specific residue interactions, rather than broad conformational shifts, underpin the altered specificity of ProTx2 mutants toward hNav1.7 over hNav1.4. Crucially, R13D’s enhanced analgesic efficacy, paired with reduced muscle toxicity, underscores the potential of targeting specific residues to improve therapeutic profiles. This nuanced understanding of peptide–channel interactions points a new direction for refining the specificity and safety of Nav1.7-targeted analgesics.

### 3.5 Distinct residue contributions elevate ProTx2 affinity to hNa_v_1.7

Free energy decomposition analysis (EDA) revealed that the significant decrease in total binding free energy (ΔEtotal) for R13D when bound to hNav1.7 highlighted a notable improvement in affinity primarily due to enhanced van der Waals and electrostatic interactions. Intriguingly, this enhancement stemmed from a cascade of effects (Table 1) initiated by the single-point mutation at R13D, which did not directly interact with hNav1.7 but significantly amplified the peptide’s affinity.

**TABLE 1:** Binding free energy of ProTx2 and its mutants to hNa_v_1.7 and hNa_v_1.4 complexes.

Complex	VDWAALS	EEl	EGB	ESURF	ΔE_total_
hNa_v_1.7-WT	−47.79	−220.48	229.46	−6.60	−45.40
hNa_v_1.7-R13A	−54.12	−252.01	257.02	−8.08	−57.19
hNa_v_1.7-R13D	−71.24	−277.09	280.69	−10.12	−77.76
hNa_v_1.7-K27Y	−58.28	−241.29	252.18	−8.54	−55.92
hNa_v_1.4-WT	−39.40	−257.41	278.09	−6.08	−24.79
hNa_v_1.4-R13A	−49.77	−347.62	375.02	−7.57	−29.94
hNa_v_1.4-R13D	−42.34	−295.34	324.62	−6.17	−19.22
hNa_v_1.4-K27Y	−51.78	−280.50	311.00	−7.47	−28.75

Energy is expressed in units of kcal/mol. VDWAALS: van der Waals interaction energy; EEL, electrostatic interaction energy; EGB, polar solvation energy; ESURF, non-polar solvation energy; ΔEtotal, total binding free energy.

The effect of the single-point mutation was first propagated through the intramolecular hydrogen bond network as D13-D10-S11-K14-Q3-C16 on mutant R13D (Supplementary Figure S6), which was called point-to-surface. This network formed an extremely stable intramolecular 3D architecture, resulting in the formation of a strong steric hindrance between C16 and K27. Predictably, K27 was the point “with wide-ranging implications.”

**FIGURE 6:**
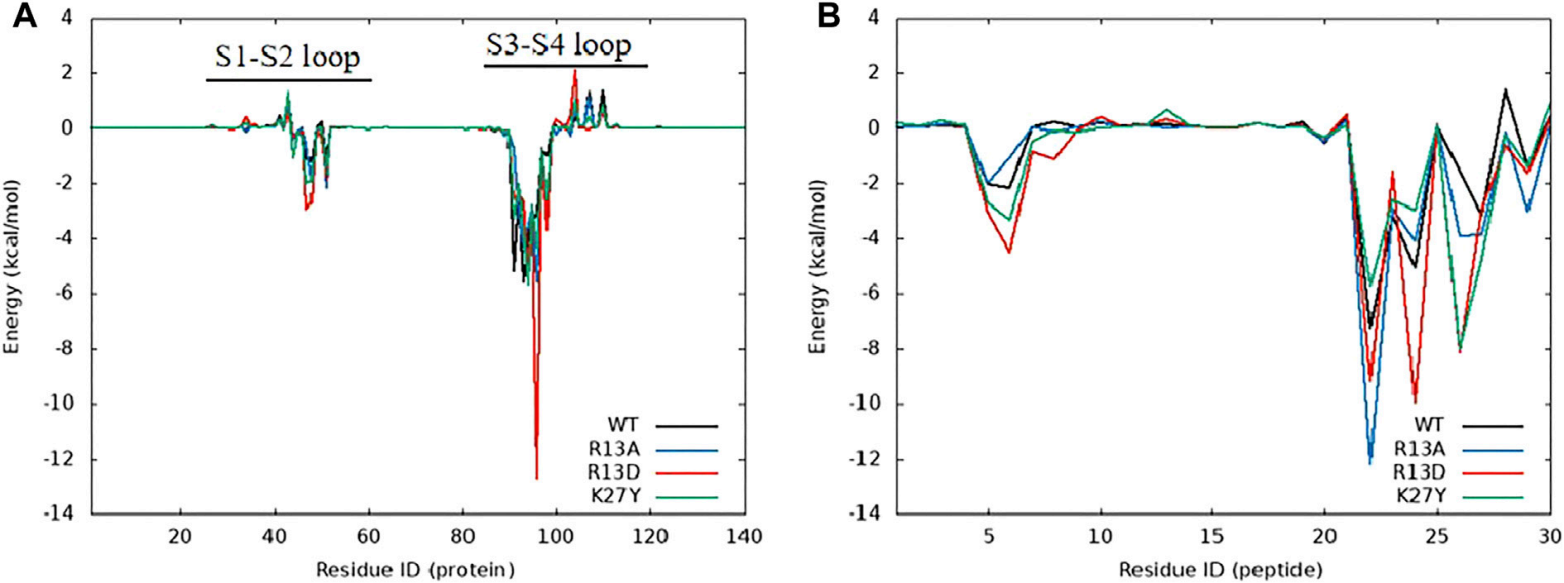
Energy contribution of amino acids in the complex system of ProTx2 and mutants with hNa_v_1.7, **(A)** hNa_v_1.7, and **(B)** ProTx2 and mutants.

**FIGURE 7:**
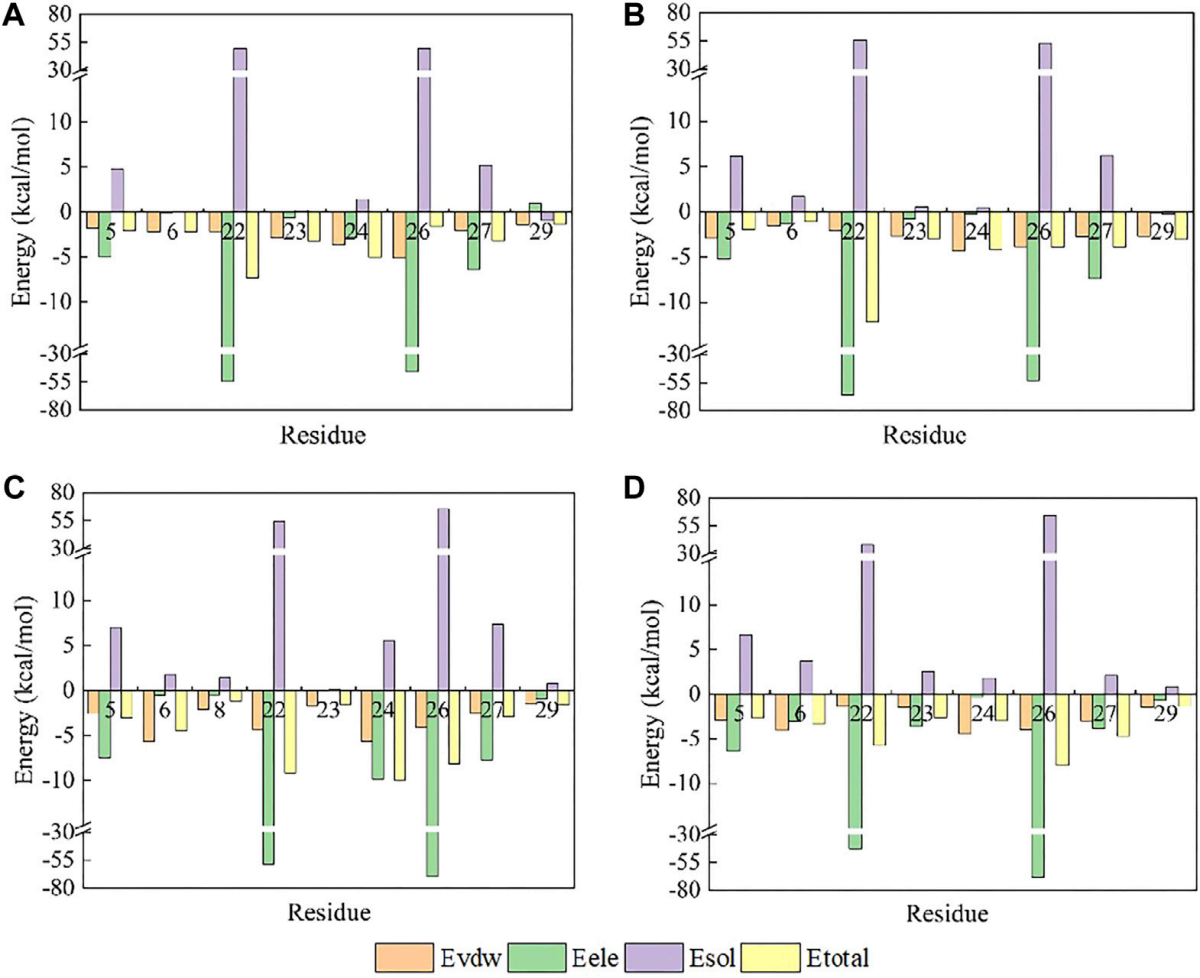
Amino acid energy decomposition diagram on ligands with outstanding energy contribution in complex systems of wild-type ProTx2 and its mutants with hNa_v_1.7: **(A)** wild-type, **(B)** R13A, **(C)** R13D, and **(D)** K17Y. Three branches of energy and total energy were colored with different columns, as shown in the picture. Two breaks were set in each system, which means the sections from 15 to 30 and from −30 to −15 were folded to present the tiny gaps among all objects.

**FIGURE 8:**
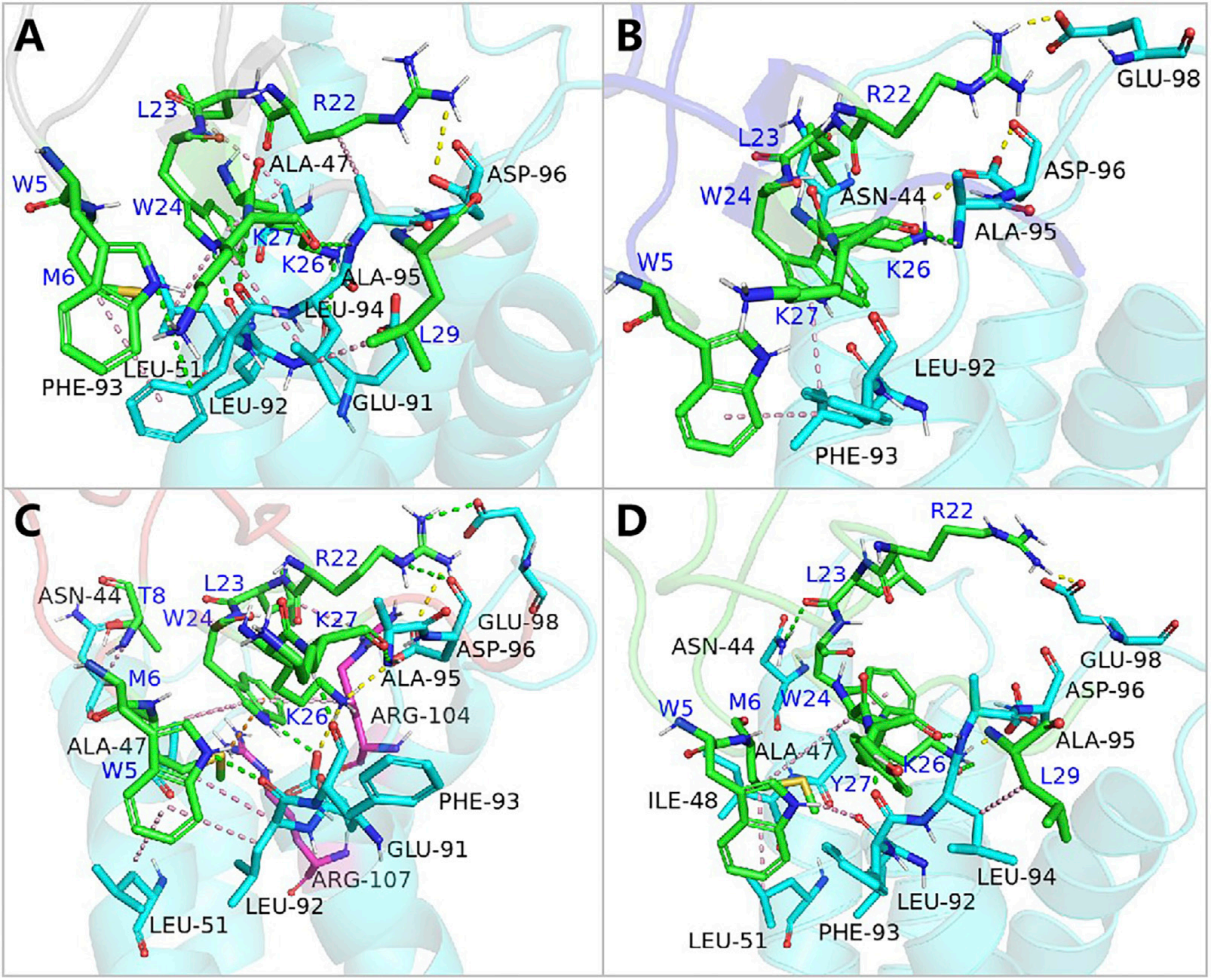
Binding poses of hNa_v_1.7 to ProTx2 and its mutants: **(A)** wild-type, **(B)** R13A, **(C)** R13D, and **(D)** K17Y. The green sticks represent the amino acids on the ligand, and the blue and purple sticks test receptor amino acids; salt bridges are marked with yellow dashed lines, hydrogen bonds with green dashed lines, hydrophobic bonds with pink dashed lines, and pi-cation with orange dashed lines.

Further analysis pinpointed K27’s pivotal role in this affinity enhancement. Adjustments in K27’s spatial positioning dramatically increased K26’s proximity to ASP96 on hNav1.7, forming a new, potent salt bridge absent in the wild-type ProTx2. This strategic mutation at site 13 thus effectively concentrated the binding energy on K26, manifesting as a surface-to-point amplification in affinity. To decipher the role of K27 in the combination of R13D and hNa_v_1.7, ΔEtotal was decomposed into each residue on the receptor and ligand in order to identify the individuals with the greatest contributions in the combination. The similar fluctuation regions (Figure 6) indicated that the binding modes of ProTx2 and its mutants to hNa_v_1.7 were almost identical, which was consistent with the analysis of DCCM and 3D models. However, it was evident that the contribution of M6 in the N-terminal and R22/W24/K26 in the C-terminal on the peptide was significantly distinct when they were located on different ligands (Table 2). Among them, the affinity increase in K26 in the mutant R13D was the most obvious as five times in contrast with wild-type ProTx2 *versus* two times with other residues. With the further EDA of the interaction type of the critical residues on the ligand in each system (Figures 7A, 8A), it was found that the major reason was that the spatial position adjustment of K27 affected K26 to be closer to ASP96 on hNa_v_1.7 and formed a new powerful salt bridge between them, which did not exist in the wild-type ProTx2 system. So far, the energy caused by the mutation at site 13 was concentrated on K26, which was called surface-to-point. Another notable difference was the newly developed interactions between W24 and two highly conserved negatively charged residues ARG104/ARG107, which were identified as the direct factors related to the activation of Na_v_s (Hampl et al., 2016). In addition, the presence of the salt bridge formed between R22 and GLU98 brought out the improvement in the contribution of R22.

**TABLE 2:** Energy contribution of amino acid residues of ProTx2 and its mutants.

Region	ProTx2 residue	hNa_v_1.7				hNa_v_1.4			
		WT	R13A	R13D	K27Y	WT	R13A	R13D	K27Y
N-terminal	Y1	-	-	-	-	−4.87	-	-	-
Irregular coil	K4	-	-	-	-	−1.8	-	-	-
Irregular turn	W5	−2.02	−1.98	−3.04	−2.7	−2.03	−4.26	-	−1.34
	M6	−2.18	−1.09	−4.49	−3.34	−1.21	−2.93	−1.39	−1.69
Irregular coil	W7	-	-	-	-	−4.9	-	-	−1.65
	T8	-	-	−1.14	-	-	-	-	−1.23
	D10	-	-	-	-	-	−1.22	-	-
	S11	-	-	-	-	-	−1.39	-	-
	X_1_13	-	-	-	-	−1.19		-	-
β-sheet	V20	-	-	-	-	-	-	-	3.3
	C21	-	-	-	-	-	-	-	−1.9
β-turn	R22	−7.25	−12.18	−9.17	−5.72	-	-	−1.74	-
	L23	−3.18	−2.92	−1.56	−2.62	-	−4.59	−1.28	−2.41
	W24	−5.04	−4.12	−9.93	−2.96	-	−2.81	−3.47	−3.05
β-sheet	K26	−1.59	−3.91	−8.15	−8	-	−3.91	−1.19	−2.6
	X_2_27	−3.17	−3.89	−2.93	−4.72	-	−2.13	-	-
	L29	−1.35	−3.03	−1.64	−1.39	-	−2.89	−3.61	−1.93

Energy is represented in units of kcal/mol. The residues at the mutation sites are labeled as X1 and X2.

While R13D emerged as a formidable variant in enhancing hNav1.7 affinity, R13A also displayed a notable increase in energy contribution from R22, leading to a stronger hNav1.7 binding compared to wild-type ProTx2 (Table 2). This was attributed to a newly formed salt bridge between R22 and GLU98 (Figures 7B, 8B). The collective data underscore R13D’s standout role in augmenting ProTx2’s affinity for hNav1.7, attributed to a synergistic interplay among key residues rather than mere conformational changes, setting a new paradigm in the specificity enhancement of peptide-based therapeutics.

Obviously, for the binding affinity between the peptide and hNa_v_1.7, the allied heroes in the mutant R13D were more intimidating than the lone hero in mutants R13A and K27Y.

### 3.6 N-terminal’s crucial role in ProTx2 binding to hNa_v_1.4

**FIGURE 9:**
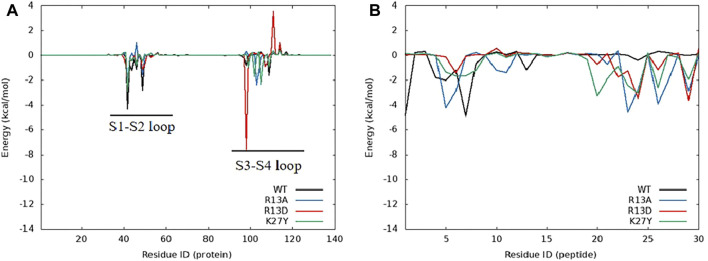
Amino acid energy contribution of the ProTx2 mutant and hNa_v_1.4 complex system: **(A)** hNa_v_1.4 and **(B)** ProTx2 mutants.

**FIGURE 10:**
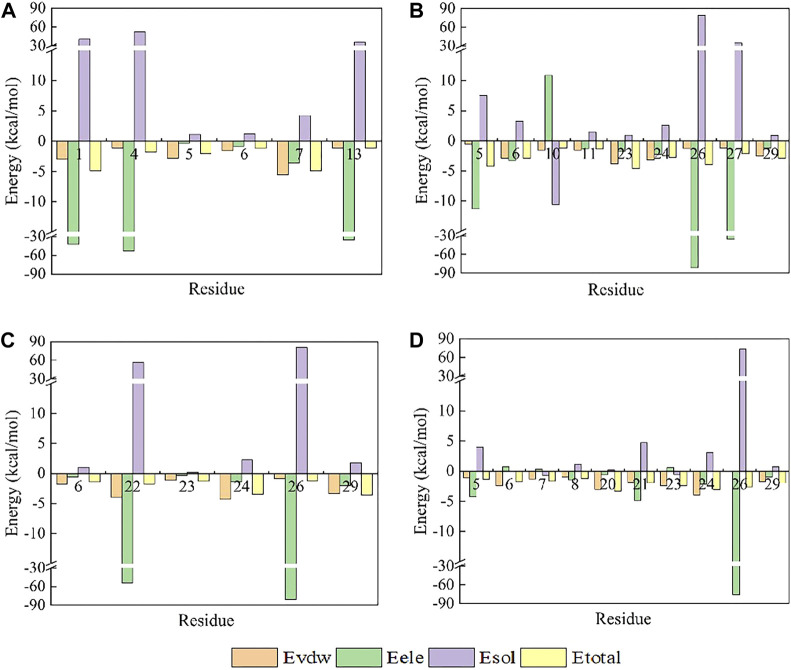
Amino acid energy decomposition diagram on ligands with outstanding energy contribution in complex systems of wild-type ProTx2 and its mutants with hNa_v_1.4: **(A)** wild-type, **(B)** R13A, **(C)** R13D, **(D)** K17Y. Three branches of energy and total energy were colored with different columns, as shown in the picture. Two breaks were set in each system, which means the sections from 15 to 30 and from −30 to −15 were folded to present the tiny gaps among all objects.

**FIGURE 11:**
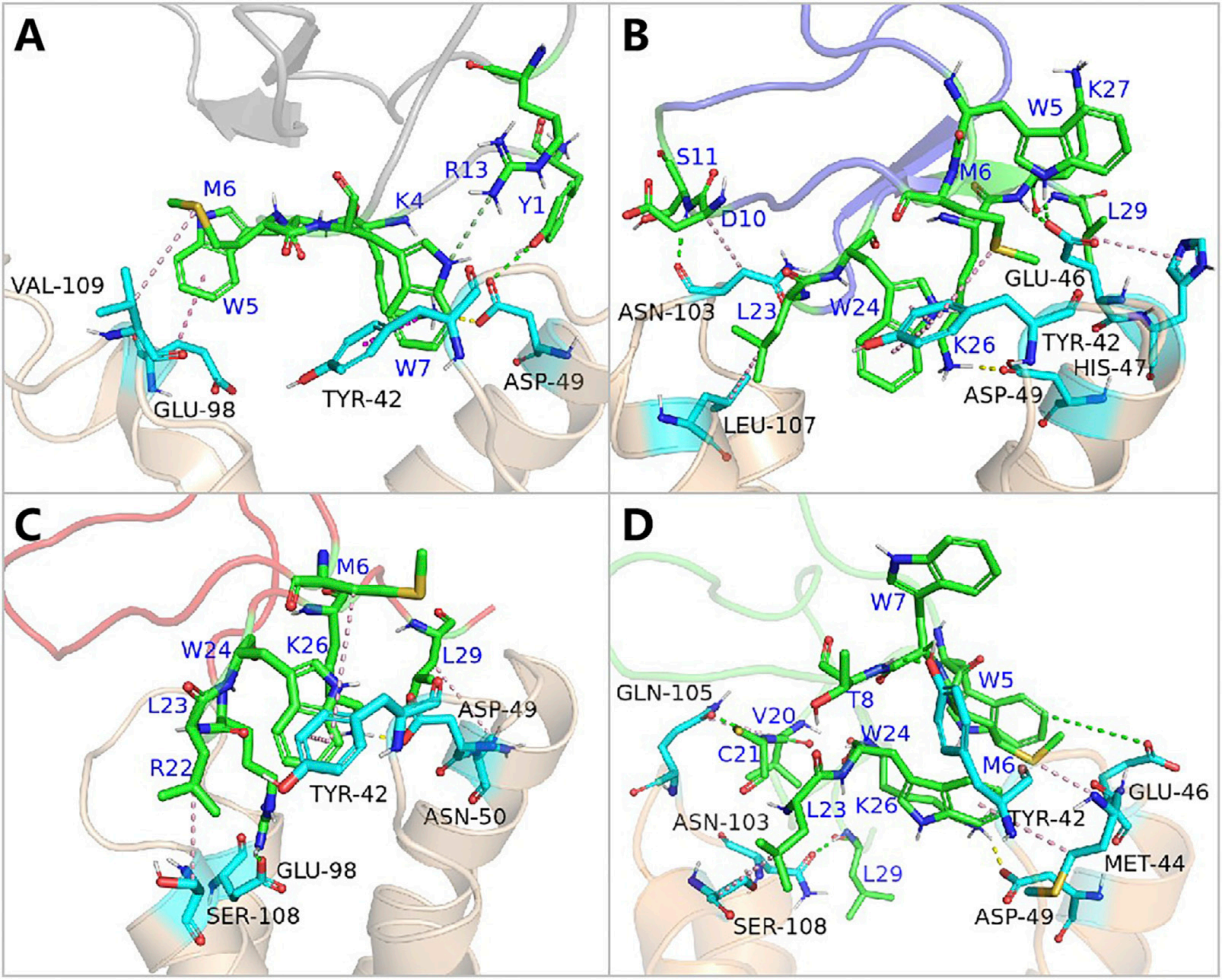
Binding pattern of hNa_v_1.4 to ProTx2 and its mutants: **(A)** wild-type, **(B)** R13A, **(C)** R13D, and **(D)** K17Y. The green sticks represent the amino acids on the ligand, and the blue indicates the receptor’s amino acids; salt bridges are marked with yellow dashed lines, hydrogen bonds with green dashed lines, hydrophobic bonds with pink dashed lines, and pi-cation with orange dashed lines.

Contrary to its interaction with hNav1.7, the total binding free energy (ΔEtotal) of ProTx2 mutants remained similar to the wild-type when bound to hNav1.4 (Table 1). However, our analysis revealed significant shifts in their binding dynamics (Figure 9). The N-terminal region, initially the primary binding site in wild-type ProTx2 (Figures 10A, 11A), underwent notable changes in its interaction pattern with hNav1.4 post-mutation, especially for R13D, where a pronounced shift toward C-terminal interactions was observed (Table 2; Figure 11C).

The alteration in R13D’s electrical properties at residue 13 eliminated its electrostatic interaction with TYR42 on hNav1.4, transforming the N-terminal’s attractive forces into repulsive forces (Figure 11C). This shift led to an increased affinity of the peptide’s C-terminal for the receptor, a pattern also seen in R13A, albeit with the N-terminal still engaging due to the neutrality of glycine at position 13 (Figures 10B, 11B). Remarkably, the mutation at residue 27 significantly influenced the binding dynamics. For instance, substituting lysine with tyrosine at this position increased the hydrophobicity of the peptide’s C-terminal, drawing K26 closer to ASP49 on hNav1.4 and forming a critical salt bridge. This interaction underscores the direct impact of mutations at site 27 on the peptide’s binding affinity (Figure 11D).

Overall, our findings highlight the N-terminal as a pivotal domain in the ProTx2’s interaction with hNav1.4, with mutations at site 13 inducing indirect effects that modulate the peptide receptor affinity. The alterations in the peptide’s binding modes, particularly the enhanced role of the C-terminal in the mutant R13D, mark a significant departure from the wild-type interactions, emphasizing the nuanced influence of specific residues on the binding efficacy.

### 3.7 Impact of the VSD2 primary structure on ProTx2 selectivity

Energy decomposition analysis (EDA) (Figures 6A, 9A) highlighted the critical role of the extracellular loops connecting S1–S2 and S3–S4 on the voltage-sensor domain 2 (VSD2) in Nav channels, identified as Loop1–2 and Loop3–4, in mediating ProTx2’s interaction with hNav1.4 and hNav1.7. Despite an 85.71% sequence similarity in the VSD2 region of these subtypes, Loop3–4’s composition diverges significantly (blue box in Supplementary Figure S7) with hNav1.7 featuring acidic residues that form strong salt bridges with R22 and K26 of ProTx2, an interaction absent in the neutral Loop3–4 of hNav1.4. This distinction enhances the binding affinity to hNav1.7 while constraining the ligand’s interaction modes, particularly weakening its engagement with Loop1–2 in hNav1.7 compared to hNav1.4. Notably, the R13D mutant exploits these structural disparities, disrupting its bond with Loop1–2 in hNav1.4 while bolstering connections with both loops in hNav1.7, as deduced from DCCM analysis (Supplementary Figure S5C; Supplementary Figure S6C).

### 3.8 Electrophysiological validation of subtype selectivity

**FIGURE 12:**
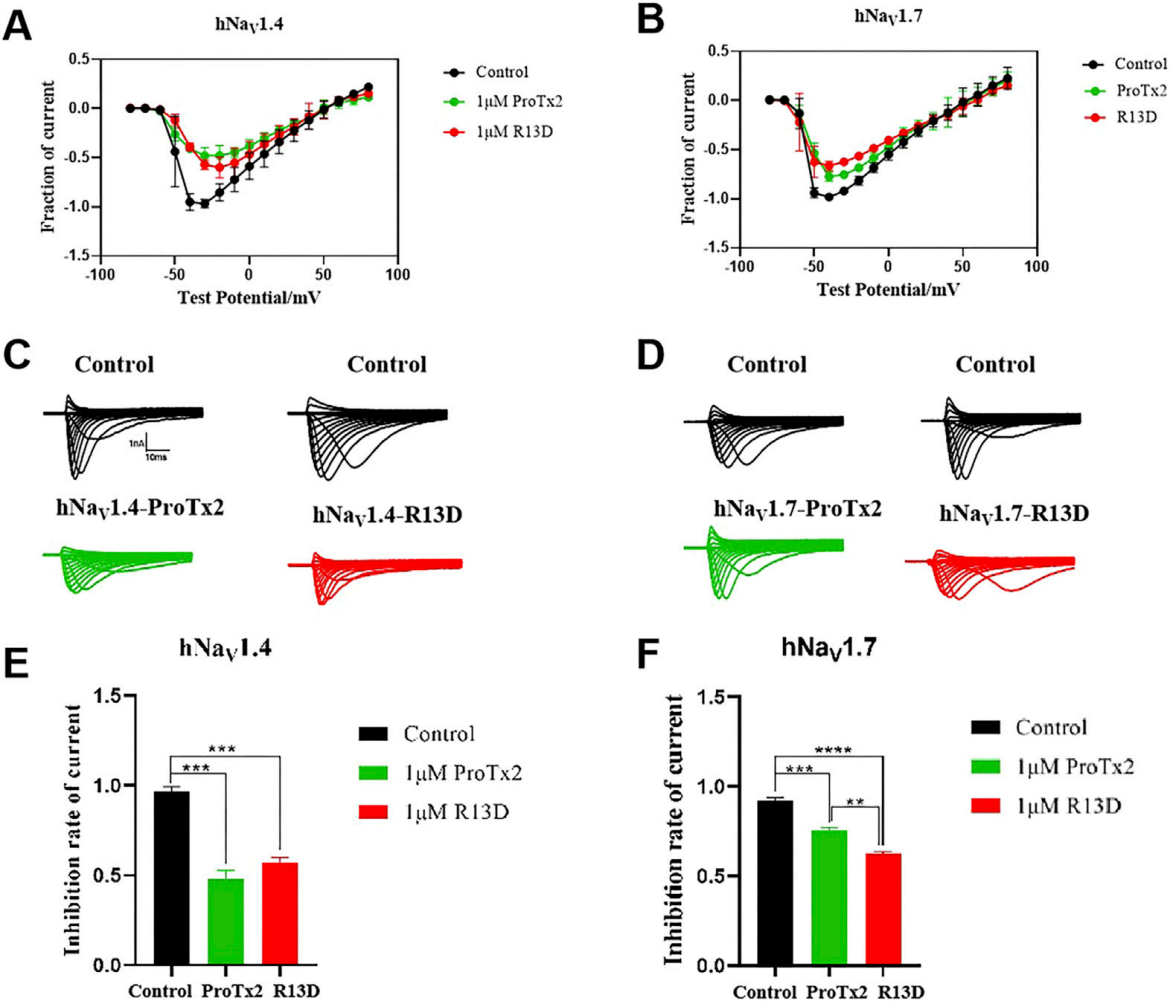
Effects of WT ProTx2 and R13D on the Na + peak current of hNa_v_1.4-CHO and hNa_v_1. 7-CHO. **(A, B)** Current–voltage relationships of hNa_v_1.4 and hNa_v_1.7. **(C, D)** The current traces were obtained from hNa_v_1.4 and hNa_v_1.7 cells in each group. **(E, F)**, Normalized Na + peak currents were depolarized at − 30 mV. (One-way ANOVA followed by Tukey’s *post hoc* tests, **p < 0.01, ***p < 0.001, and ****p < 0.0001).

Electrophysiological assays quantified the effects of ProTx2 and its R13D mutant on sodium currents within CHO cells expressing hNav1.4 and hNav1.7 (Figure 12). Although both ProTx2 and R13D inhibited currents across these subtypes, R13D exhibited a marginally diminished effect on hNav1.4, yet pronouncedly intensified inhibition of hNav1.7, aligning with predictions from molecular dynamics simulations. Specifically, at 1 μM concentration, R13D notably decreased the peak sodium current amplitude in hNav1.7 from 82% to 68% (*p* < 0.01) and modestly increased it in hNav1.4 from 50% to 59%. This shift underscores R13D’s enhanced analgesic potential and reduced muscle toxicity, corroborating the insights gained from computational and experimental analyses.

## 4 Discussion

ProTx2 has attracted attention due to its exceptional inhibitory effectiveness against Na_v_1.7. However, it also exhibits potent inhibition of other Nav family members, such as the off-targets Na_v_1.4 and Na_v_1.5 (Cardoso and Lewis, 2019). In earlier studies, the SAR research on ProTx2 over Na_v_1.5 was investigated, and some analogs were designed to illustrate the important role of several positively charged residues such as R13, R22, and K27 (Smith et al., 2007). In 2019, the revelation crystal structure further elucidated the interaction between ProTx2 and Na_v_1.7 (Xu et al., 2019). However, the mechanism of this spider toxin to Nav1.4, as the main channel for signal transmission in muscles, is rarely reported. This undoubtedly poses a potential risk for the development of ProTx2 as a potential analgesic. In this study, the enhanced analgesic effect of the R13D mutant without the accompanying muscle toxicity represents a significant advancement in the development of safer pain management therapies. The study’s integration of molecular dynamics and electrophysiological data provides a robust framework for understanding the interactions between peptide toxins and voltage-gated sodium channels at a granular level. Notably, the discovery that specific residues contribute more decisively to the peptide’s binding affinity and subtype selectivity than overall structural changes offers valuable insights for the design of targeted therapeutics.

**FIGURE 13:**
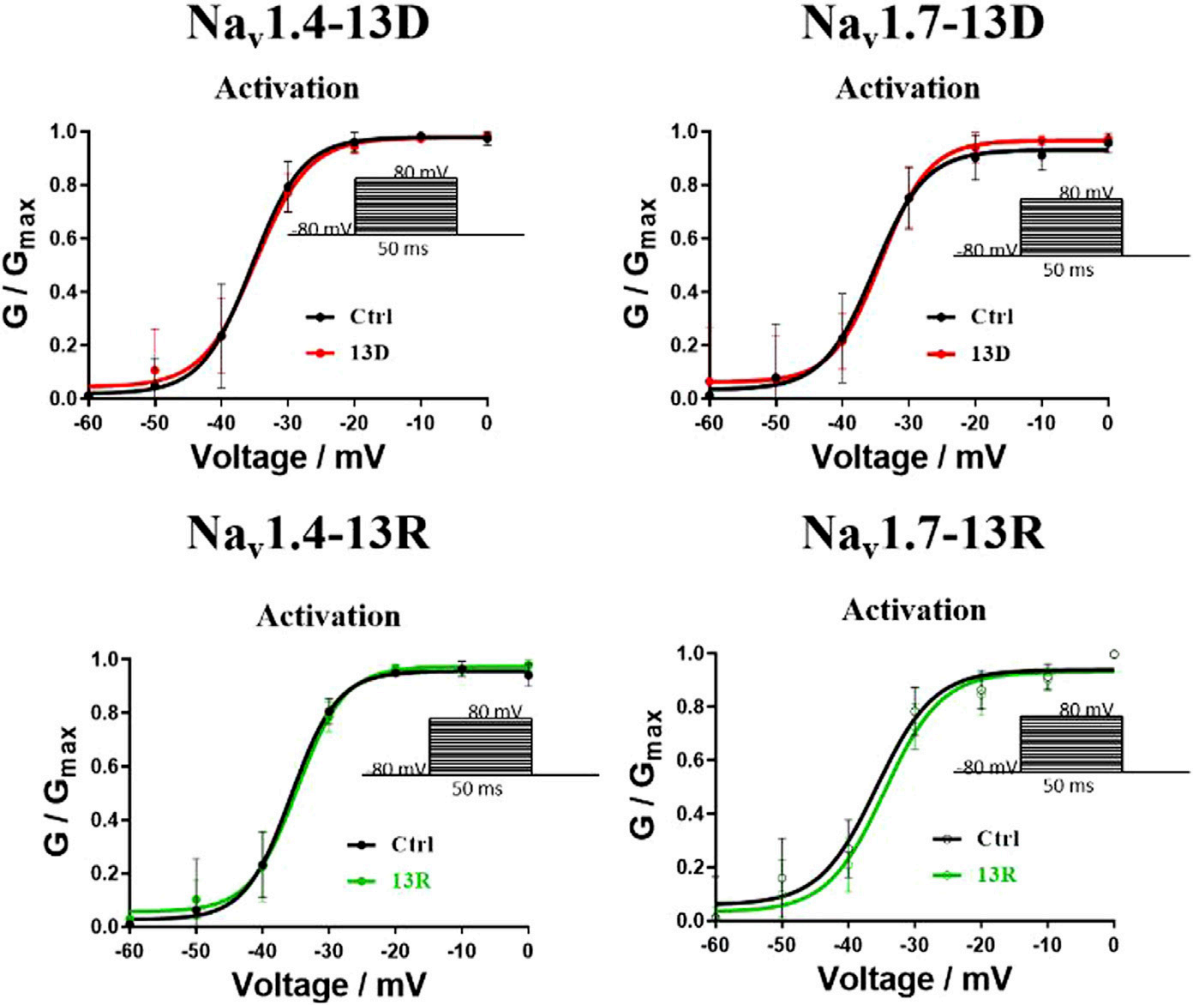
Effects of ProTx2 on voltage-dependence activation of Nav1.4 and Nav1.7.

However, this study is not without limitations. Although the *in vitro* findings are promising, the *in vivo* efficacy and safety of the R13D mutant remain to be fully established. The long-term effects, potential immunogenicity, and pharmacokinetics of the R13D peptide in a physiological context warrant further investigation. Additionally, the study focuses on a single mutation, and the interplay between multiple mutations and their cumulative effects on the peptide’s properties could offer further opportunities for optimization. On the other hand, in the presence or absence of ProTx2, the inactivation of the two subtypes Na_v_s remained essentially unchanged, while the activation showed slight differences according to the results of the whole-cell clamp patch. However, no significant differences were observed as well in the analysis of activation curves (Figure 13). Given the inhibition of the peak current of Na_v_s from ProTx2, it was deduced that the structural restrictions on the VSDs ultimately manifested as blockages of the change in pore domains. Nevertheless, there were no explanations about the molecular mechanism underlying this association.

Future research should aim to validate the therapeutic potential of the R13D mutant in animal models of pain and explore the possibility of combining multiple beneficial mutations to further enhance the peptide’s specificity and efficacy. Moreover, expanding the scope of the study to include other sodium channel subtypes could provide a more comprehensive understanding of the peptide’s selectivity profile and its implications for therapeutic development.

## 5 Conclusion

This study provides compelling evidence that the R13D mutant of ProTx2 exhibits enhanced specificity and reduced toxicity compared to the wild-type peptide, establishing it as a promising candidate for the development of non-addictive analgesics targeting the hNav1.7 channel. Molecular dynamics simulations and electrophysiological assays reveal that the altered interaction patterns of ProTx2 mutants, particularly R13D, with hNav1.7 and hNav1.4 are largely due to specific residue modifications rather than broad conformational changes. These findings underscore the critical role of the N-terminal domain in binding affinity and highlight the influence of VSD2’s primary structure on the subtype selectivity of ProTx2.

In terms of binding modes, ProTx2 and its mutants exhibited similar binding patterns with hNa_v_1.7 while showing significant differences with hNa_v_1.4. Despite this, compared to the wild-type peptide, the mutants displayed significant changes in affinity with hNa_v_1.7 but not with hNa_v_1.4. The reason could be attributed to the remarkable enhancement of several individual residues on the mutations, resulting in substantial changes in affinity with hNa_v_1.7. On the other hand, the affinity between the ligand and hNa_v_1.4 was greatly influenced by the N-terminal region on the peptides.

## Data Availability

The original contributions presented in the study are included in the article/Supplementary Material; further inquiries can be directed to the corresponding author.
