# Supplementary material for: The road to evolution of ProTx2: how to be a subtype-specific inhibition of human Nav1.7
Source: Front Pharmacol. 2024 May 2;15:1374183. doi: 10.3389/fphar.2024.1374183 (PMC11096480; doi:10.3389/fphar.2024.1374183)
Supplement: Supplementary file 1 [file Image1.pdf]

Supplementary Material

1 Supplementary Figures

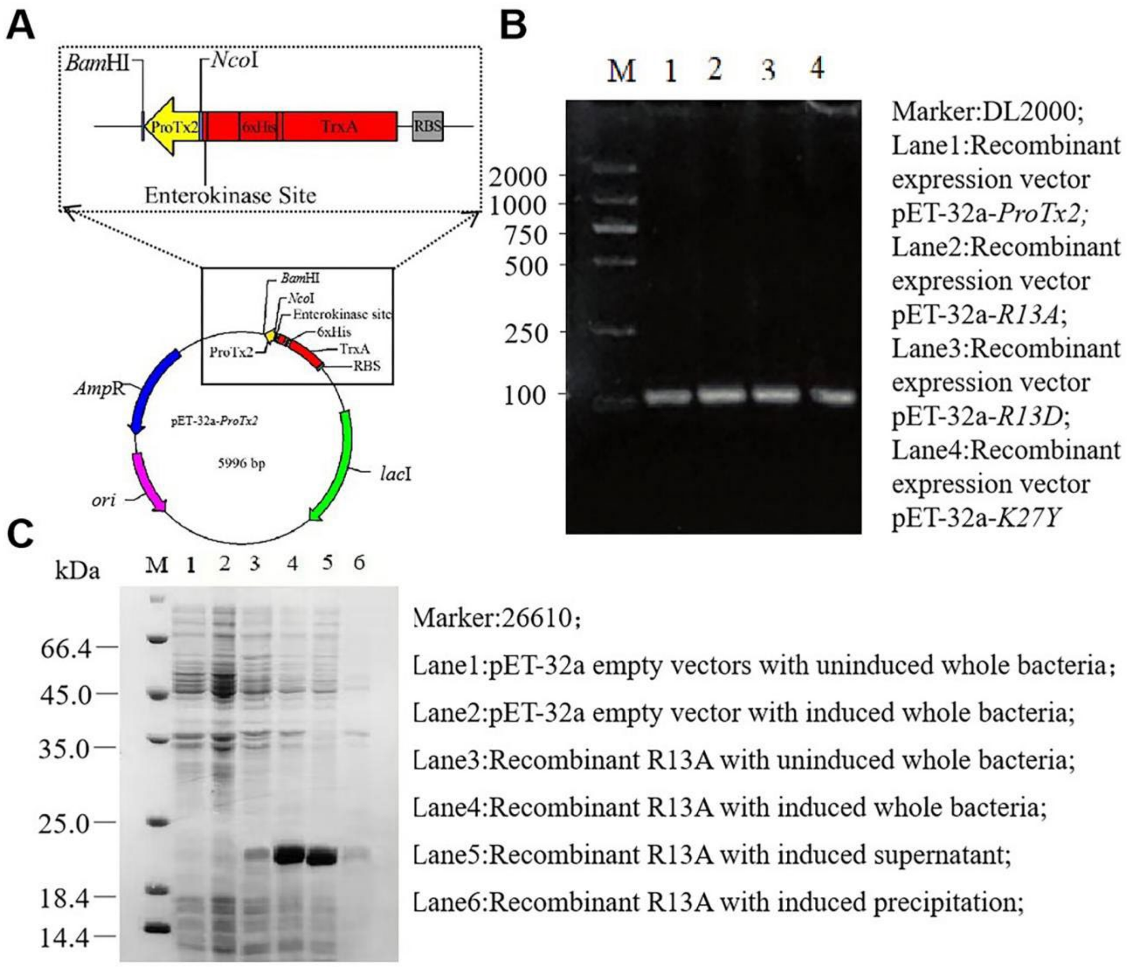

Supplementary Figure 1. Recombinant expression strategy of ProTx2 and mutants. (A) Schematic diagram of the recombinant expression vector pET-32a-ProTx2. The illustration shows the ProTx2 fusion protein containing TrxA as the solubilizing labels, 6xHis for purification and the enterokinase site for the cleavage by enterokinase; (B) 3% agarose gel electrophoresis profle displaying recombinant expression vector; (C) 12% SDS-PAGE analysis of soluble expression of recombinant R13A.

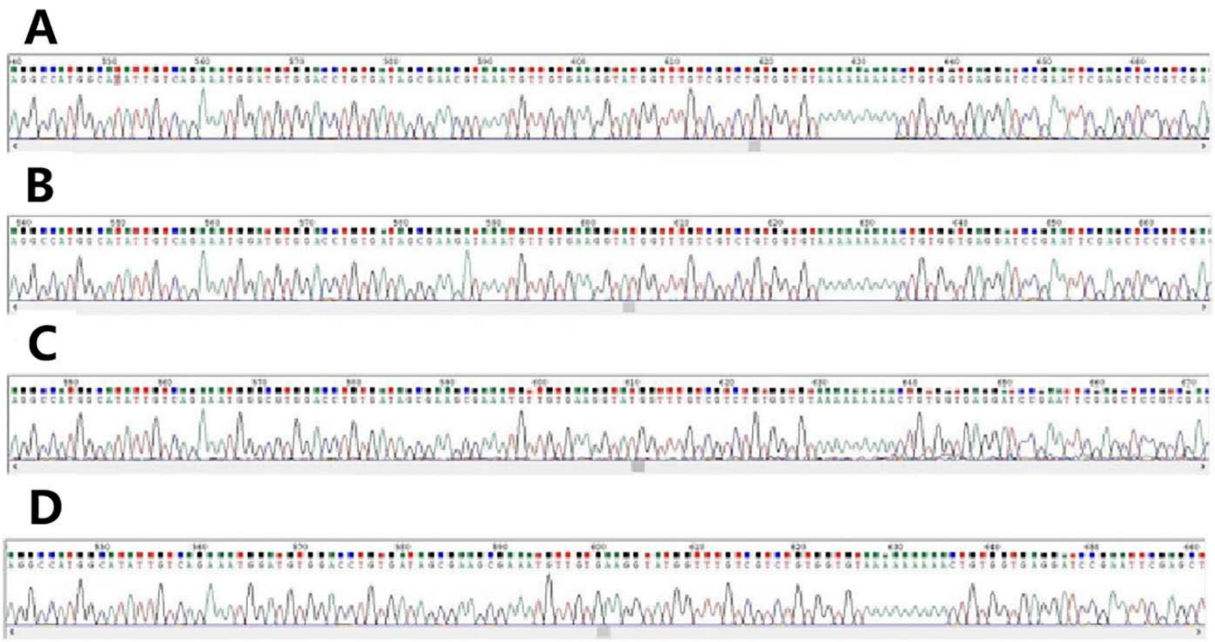
A
B
C
D

Supplementary Figure 2. Expression vector gene sequencing results of ProTx2 and its mutant protein. (A) ProTx2. (B) R13D. (C) R13A. (D) K27Y.

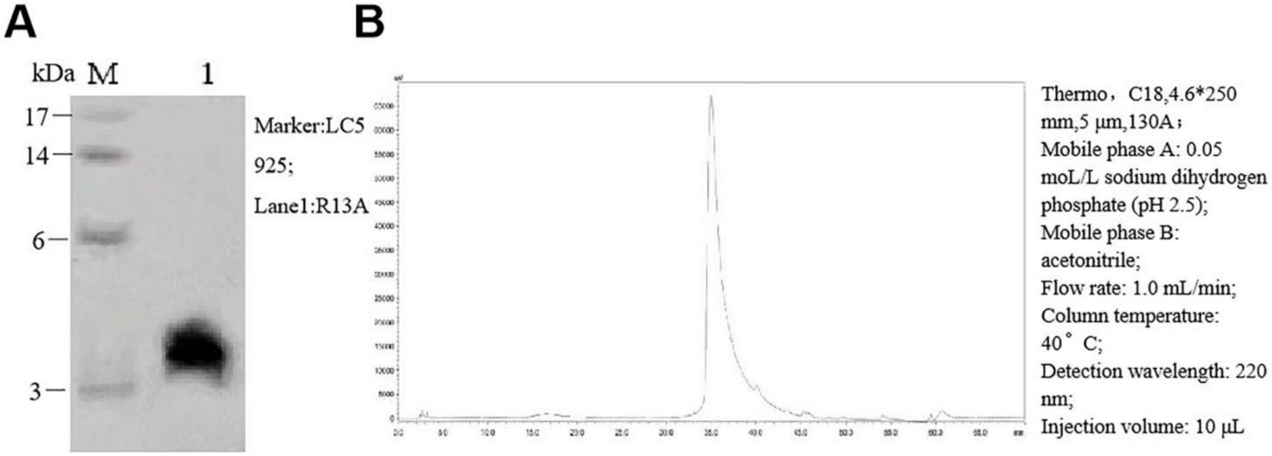

Supplementary Figure 3. Verification of ProTx2 Purity. **(A)** 20% Tricine-SDS-PAGE showing ProTx2 cleavage by enterokinase. **(B)** The protein ProTx2 profiles of High Performance Liquid Chromatographic.

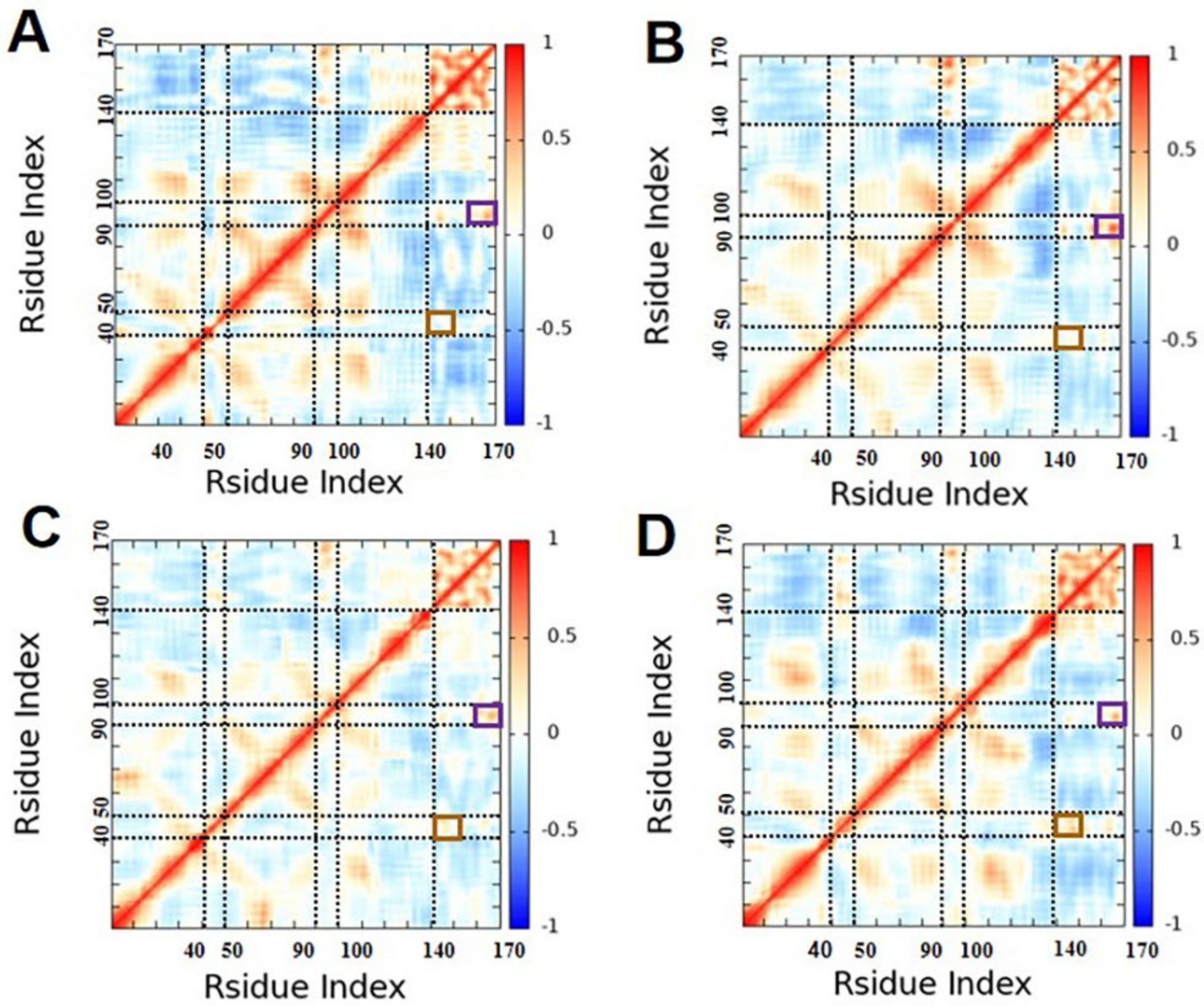

Supplementary Figure 4. DCCM of ProTx2 and its mutants with hNav1.7. **(A)** Wild-type. **(B)** R13A. **(C)** R13D. **(D)** K17Y. The horizontal and vertical coordinates represent amino acid numbers, where 1-140 residues are in DII/S1-S4 on the Navs and 141-170 residues are on the peptide.

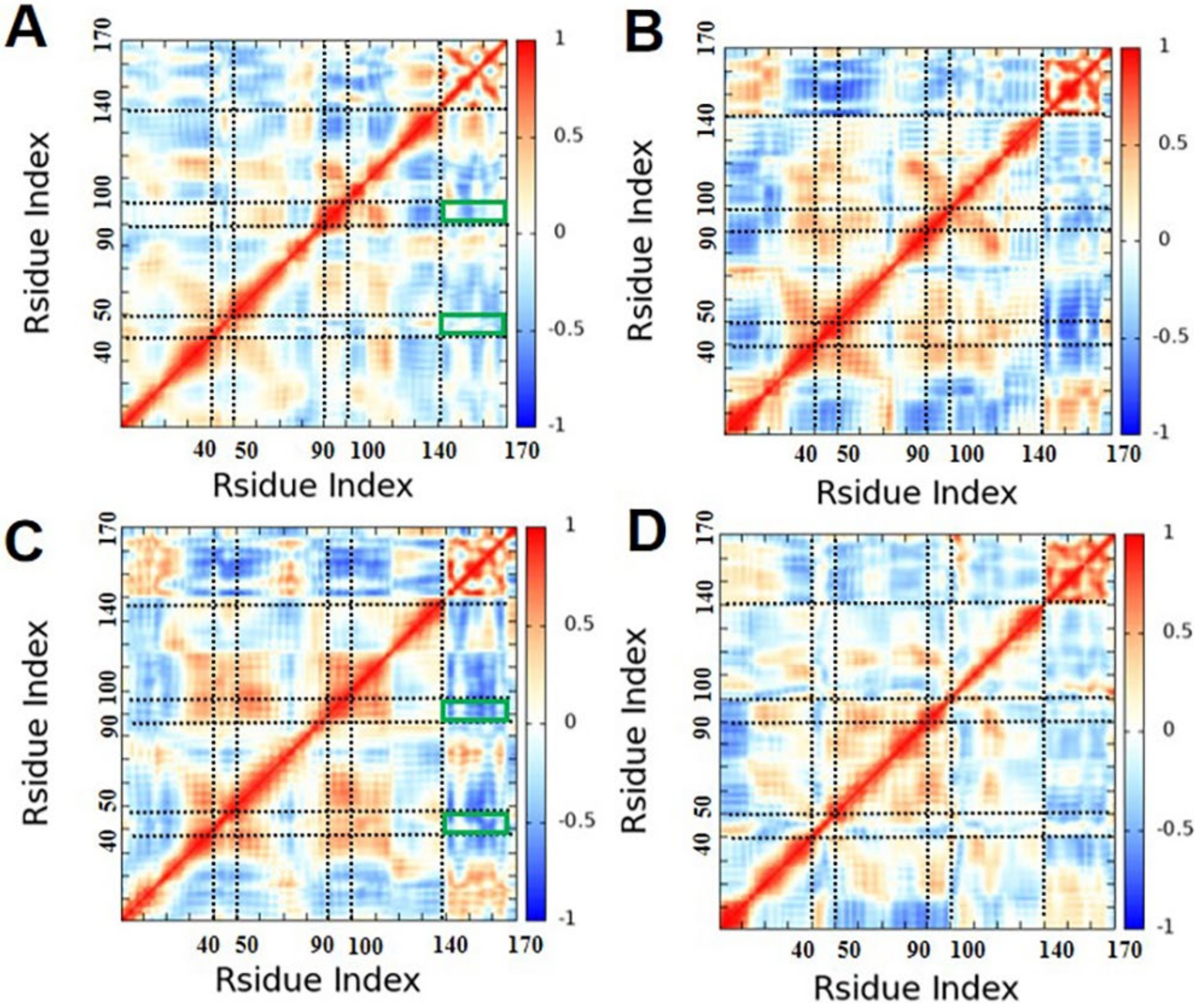

Supplementary Figure 5. DCCM of ProTx2 and its mutants with hNav1.4. **(A)** Wild-type. **(B)** R13A. **(C)** R13D. **(D)** K17Y. The horizontal and vertical coordinates represent amino acid numbers, where 1-140 residues are in DII/S1-S4 on the Navs and 141-170 residues are on the peptide.

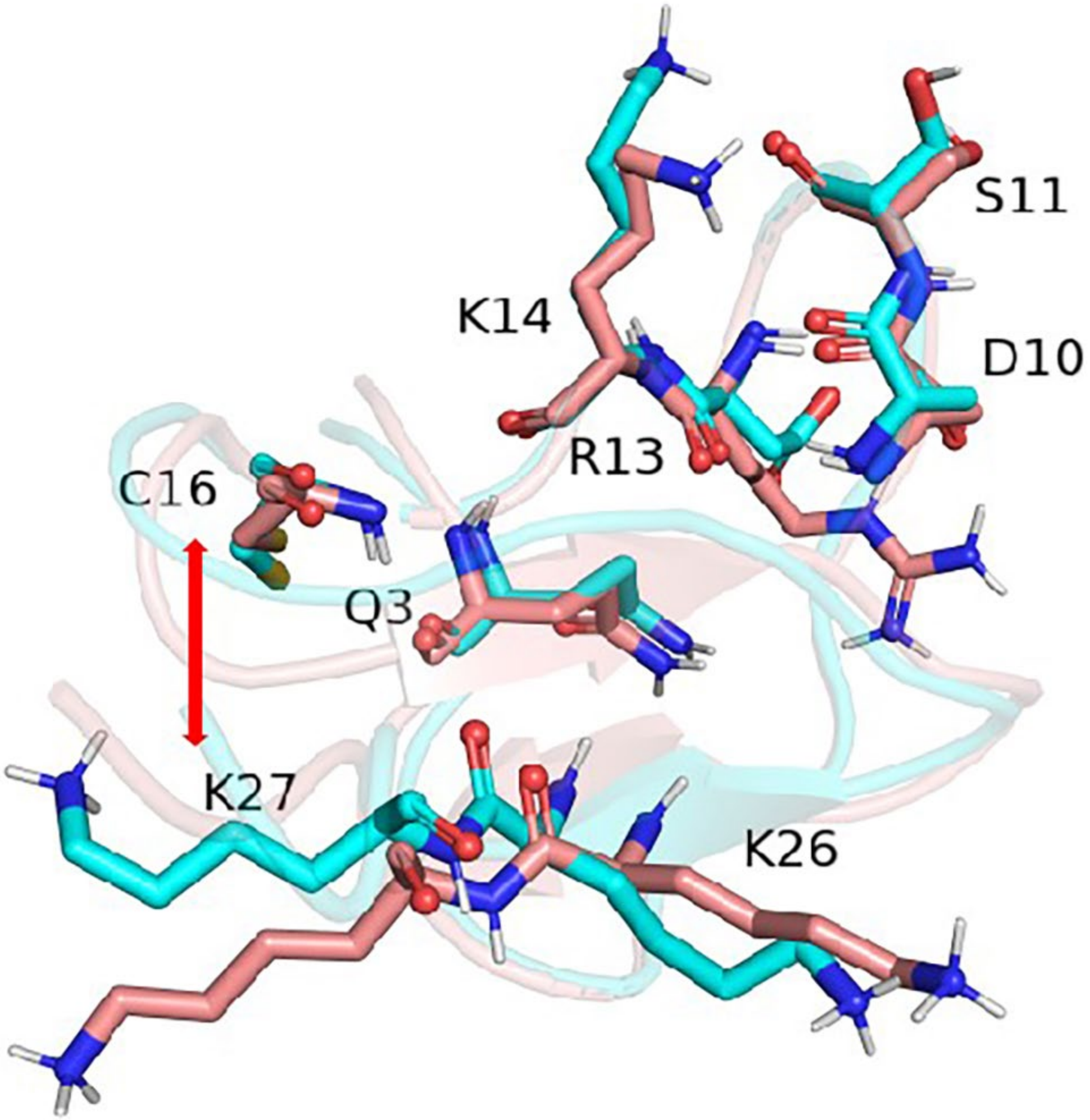

Supplementary Figure 6. Hydrogen bond Chain of significant residues in wild-type toxin and R13D mutant. Polypeptides were showed as cartoons and important residues as sticks, in which wild type was salmon and R13D was cyan. Hbond-chain was showed as arrows with green dotted lines,

indicating the directions of hbonds. Arrows with yellow were represented the position changes of K27 and K26 from wild to R13D. Red arrow showed the steric hindrance between C16 and K27.

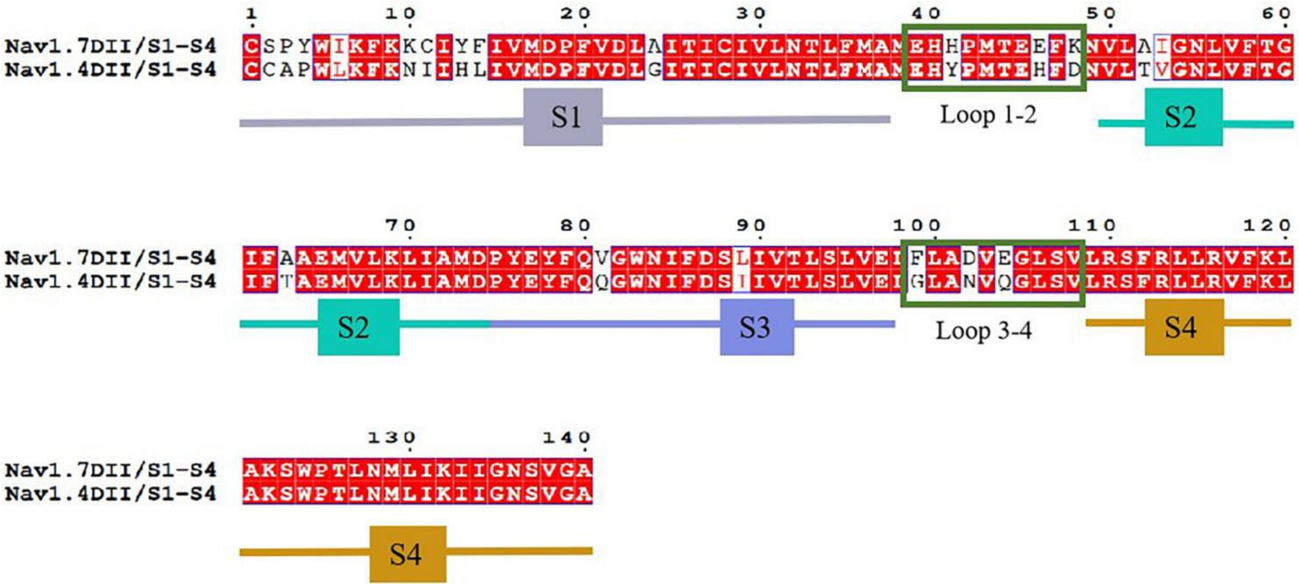

Supplementary Figure 7. Sequence alignment of hNav1.7/1.4 DII /S1-S4. In the figure, the red and white letters indicate that the amino acids are completely consistent, the red letters on the white background indicate that the amino acids are similar in nature, and the black letters on the white background indicate that the amino acids are different in nature. The number on the top of the sequence indicates the number of amino acids. S1-S4 were annotated on the bottom of sequences in

different colors and Loop 1-2 and Loop 3-4 (connection between S3-S4) were showed with green boxes.

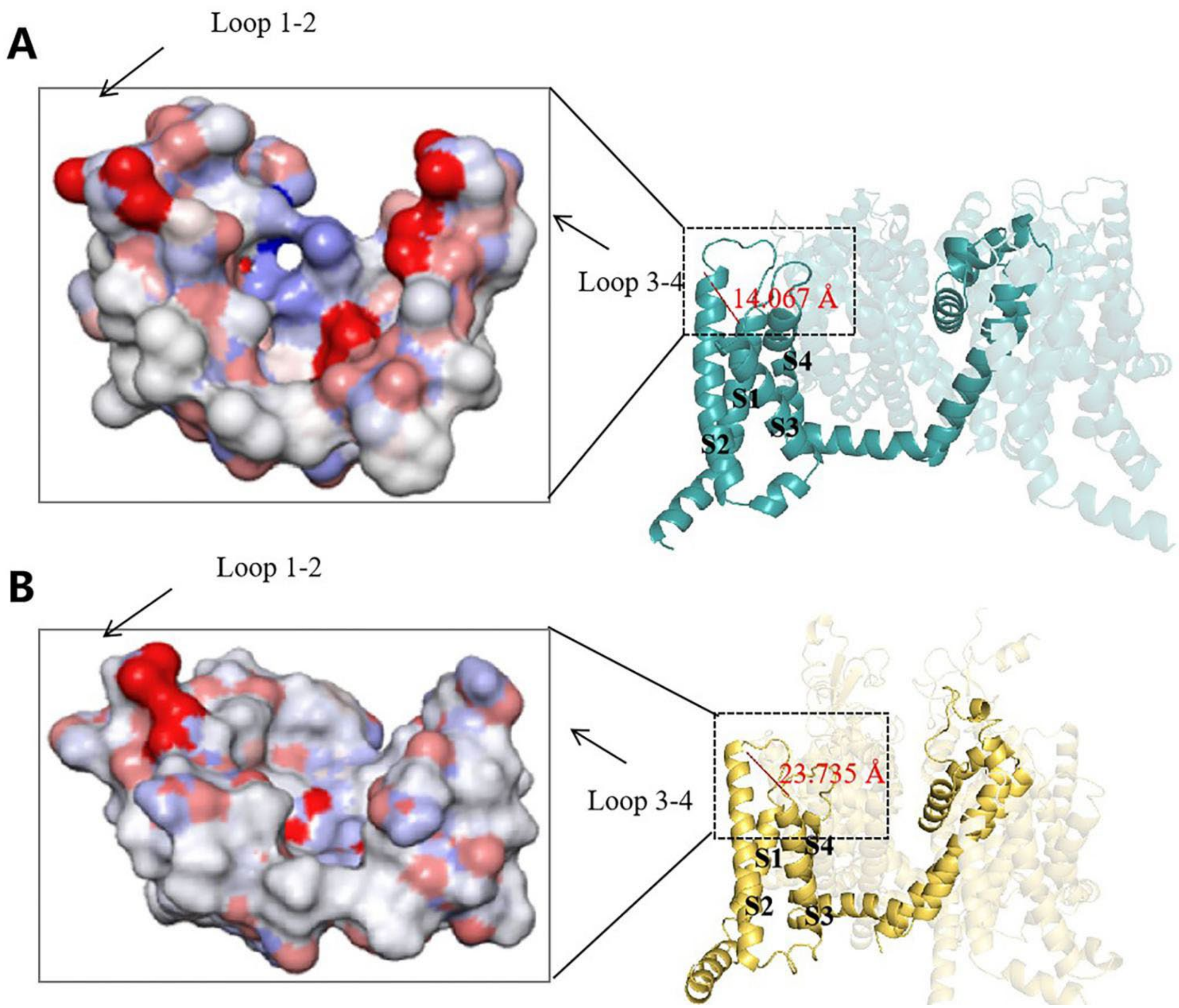

Supplementary Figure 8. Schematic diagram of the DII/S1-S4 region of hNav1.7 in A and hNav1.4 in B. The inset is an electrostatic surface diagram, where red represents the negative, blue represents the positive, and red lines and numbers represent the distance from S1 tip to S3 tip of DII region.
